# Study on the Classification Method of Rice Leaf Blast Levels Based on Fusion Features and Adaptive-Weight Immune Particle Swarm Optimization Extreme Learning Machine Algorithm

**DOI:** 10.3389/fpls.2022.879668

**Published:** 2022-05-06

**Authors:** Dongxue Zhao, Shuai Feng, Yingli Cao, Fenghua Yu, Qiang Guan, Jinpeng Li, Guosheng Zhang, Tongyu Xu

**Affiliations:** ^1^College of Information and Electrical Engineering, Shenyang Agricultural University, Shenyang, China; ^2^Liaoning Engineering Research Center for Information Technology in Agriculture, Shenyang, China

**Keywords:** rice, leaf blast, hyperspectral, fusion features, disease classification

## Abstract

Leaf blast is a disease of rice leaves caused by the Pyricularia oryzae. It is considered a significant disease is affecting rice yield and quality and causing economic losses to food worldwide. Early detection of rice leaf blast is essential for early intervention and limiting the spread of the disease. To quickly and non-destructively classify rice leaf blast levels for accurate leaf blast detection and timely control. This study used hyperspectral imaging technology to obtain hyperspectral image data of rice leaves. The descending dimension methods got rice leaf disease characteristics of different disease classes, and the disease characteristics obtained by screening were used as model inputs to construct a model for early detection of leaf blast disease. First, three methods, ElasticNet, principal component analysis loadings (PCA loadings), and successive projections algorithm (SPA), were used to select the wavelengths of spectral features associated with leaf blast, respectively. Next, the texture features of the images were extracted using a gray level co-occurrence matrix (GLCM), and the texture features with high correlation were screened by the Pearson correlation analysis. Finally, an adaptive-weight immune particle swarm optimization extreme learning machine (AIPSO-ELM) based disease level classification method is proposed to further improve the model classification accuracy. It was also compared and analyzed with a support vector machine (SVM) and extreme learning machine (ELM). The results show that the disease level classification model constructed using a combination of spectral characteristic wavelengths and texture features is significantly better than a single disease feature in terms of classification accuracy. Among them, the model built with ElasticNet + TFs has the highest classification accuracy, with OA and Kappa greater than 90 and 87%, respectively. Meanwhile, the AIPSO-ELM proposed in this study has higher classification accuracy for leaf blast level classification than SVM and ELM classification models. In particular, the AIPSO-ELM model constructed with ElasticNet+TFs as features obtained the best classification performance, with OA and Kappa of 97.62 and 96.82%, respectively. In summary, the combination of spectral characteristic wavelength and texture features can significantly improve disease classification accuracy. At the same time, the AIPSO-ELM classification model proposed in this study has sure accuracy and stability, which can provide a reference for rice leaf blast disease detection.

## Introduction

Rice is one of the world’s major food crops, widely grown in more than 100 countries worldwide. Its stable production plays an essential role in ensuring global food security and reducing rural poverty (Sharma et al., 2012). With the drastic changes in the environment and the climate in recent years, pests and diseases have occurred frequently, causing considerable losses to rice production. Rice blast is one of the major diseases affecting rice production. It is a widespread and destructive rice disease caused by the Pyricularia oryzae. Failure to prevent and control Pyricularia oryzae in a timely and effective manner will generally result in yield reductions of 10%–20%, severe cases 40%–50%, or even crop failure (Deng et al., 2017; Li et al., 2019). Rice blast can be divided into the seedling blast, leaf blast, nodal blast, and spike blast according to the damage period and damage site, among which leaf blast is the most common occurrence. Leaf blast forms poke-shaped spots on rice leaves, and the leaf tissue is severely damaged at the spot, affecting the photosynthesis of the rice plant. Meanwhile, at the heading date, the production of conidia at leaf spots will infest rice ears affecting yield and quality (Kobayashi et al., 2001). Therefore, in this study, rice leaf blast was studied to classify and identify different disease levels of rice leaf blast.

The traditional method of leaf blast detection mainly relies on agronomic experts to obtain rice disease information visually in the field based on their experience. This method depends primarily on the experience of experts, which is time-consuming, laborious, and subjective (Zhang et al., 2012). This approach is costly, time-consuming, and challenging to implement for extensive farmlands. PCR, although highly accurate in disease detection, requires specialist knowledge and equipment to identify and interpret the test results reliably (Deng et al., 2020). Therefore, there is still an urgent need for a more accurate, practical, and simple method for the early detection of leaf blast.

In contrast, digital imaging and hyperspectral imaging are widely used by scholars for disease detection and analysis. Digital imaging is more attractive for field applications due to its advantages of fast imaging, low cost, non-destructive nature, and the ability to detect diseases through various representations such as leaf shape and texture (Zhang et al., 2020). Hyperspectral imaging technology combines the advantages of spectral and machine vision techniques and contains both spectral and spatial domain information in hyperspectral images (Gu et al., 2019). Compared to digital images, hyperspectral images contain biochemical information about the leaf, inherent structural information, and leaf characterization information, making early disease detection possible. At the same time, subtle changes in spectral reflectance are complemented by changes in image characterization, making the difference between early and regular diseases more apparent. In recent years, hyperspectral imaging techniques have been widely used in research areas such as plant disease detection (Zhao et al., 2016a; Lu et al., 2018b; Feng et al., 2021; Tian et al., 2021), nutritional diagnosis (Feng et al., 2020b; Li et al., 2020a; Xu et al., 2020; Yoosefzadeh-Najafabadi et al., 2021a), and quality testing (Keresztes et al., 2016; Liu et al., 2018; Lu et al., 2018a) due to their fast, accurate, and non-destructive characteristics. And in the early detection of disease, Calamita et al. (2021) used hyperspectral techniques to extract vegetation indices, such as GDVI, MGVI, and OSAVI to construct classification models for Armillariella tabescens, such as LDA, NB, and RDA, and the results showed that the NB classification model achieved the best classification accuracy with 90% accuracy. Gao et al. (2020) screened sensitive wavelengths for Grape Leafroll disease based on variance analysis and linear regression analysis. They used the least squares support vector machine (SVM) approach to construct a classification model with an optimal classification accuracy of 93%. Fajardo et al. (2020) constructed a PLS-PLR detection model for banana black sigatoka with 98% accuracy using hyperspectral data. Gu et al. (2019) used the successive projections algorithm to get six spectral wavelengths associated with tobacco tomato spotted wilt virus (TSWV) to construct a boosted regression tree (BRT) classification model with an overall accuracy of 85.2% and an AUC of 0.932. Although the above studies also achieved good results in disease classification, most of them only used spectral features to construct models for disease identification. They did not combine the image information of the object to be detected. Spectral information alone can easily misclassify in experimental field conditions, especially in early disease detection.

There have also been some studies in diseases using spectral information and image information to identify conditions in recent years. Xie and He (2016) constructed KNN and Adaboost diagnostic models based on spectral features and texture features to classify healthy and susceptible samples of eggplant early blight, respectively. In the test set, all models achieved a classification rate of more than 88.46%. Khan et al. (2021) extracted the normalized difference texture indices (NDTIs) and vegetation indices (VIs) from hyperspectral images of wheat powdery mildew. They constructed a partial least-squares linear discrimination analysis model. The results showed that the discrimination model combining VIs and NDTIs improved the early identification of infected leaves with accuracy and kappa coefficient exceeding 82.35% and 0.56, respectively. Huang et al. (2020) investigated the early detection of Fusarium head blight on wheat spikes. They showed that the PSO-SVM model constructed based on fused features of spectra and images had the highest recognition accuracy. In the above studies, good results have been achieved in detecting diseases such as wheat and aubergine using spectral features combined with image features, but not much has been seen in the detection of rice leaf blast. Also, in spectral-based disease detection, the selection of the model method has a crucial influence on the detection accuracy.

In general, machine learning methods can perform quantitative (Yu et al., 2020; Yoosefzadeh-Najafabadi et al., 2021b) and qualitative (Liu et al., 2010; Li et al., 2020a) crop detection. However, due to the limited amount of data or insignificant differences between features, traditional machine learning methods cannot detect accurately, such as SVM and other models (Greener et al., 2022). Huang et al. (2006) proposed a neural network algorithm, the Extreme Learning Machine (ELM). ELM is regarded as a special kind of feed-forward neural network, which has a more significant advantage in learning rate and generalization than other shallow learning models. However, it was found that the ELM’s weight and bias generation approach tends to lead to problems such as instability model results and lack of interpretation of the learning process. Therefore, we use an adaptive-weight immune particle swarm optimization algorithm for ELM optimization (AIPSO-ELM). The method introduces adaptive weights into the main algorithm to avoid missing optimal solutions. At the same time, an immune particle swarm is used instead of a standard particle swarm to ensure particle diversity and effectively prevent premature convergence. This AIPSO-ELM model approach has not previously been used in the early detection of rice leaf blast in hyperspectral images.

In view of this, the overall research objective of this study was to construct a model for the identification of different grades of rice leaf blast using spectral features combined with texture features. The specific research objectives are: (1) to determine the sensitive spectral wavelengths associated with rice leaf blast, (2) to extract texture features from hyperspectral images of rice leaves, and to construct fusion features by combining the obtained texture features with the filtered feature wavelengths, and (3) to propose a disease class classification method and compare and analyze it with other modeling methods to determine the best classification model.

## Materials and Methods

### Study Area and Experimental Details

**Figure 1:**
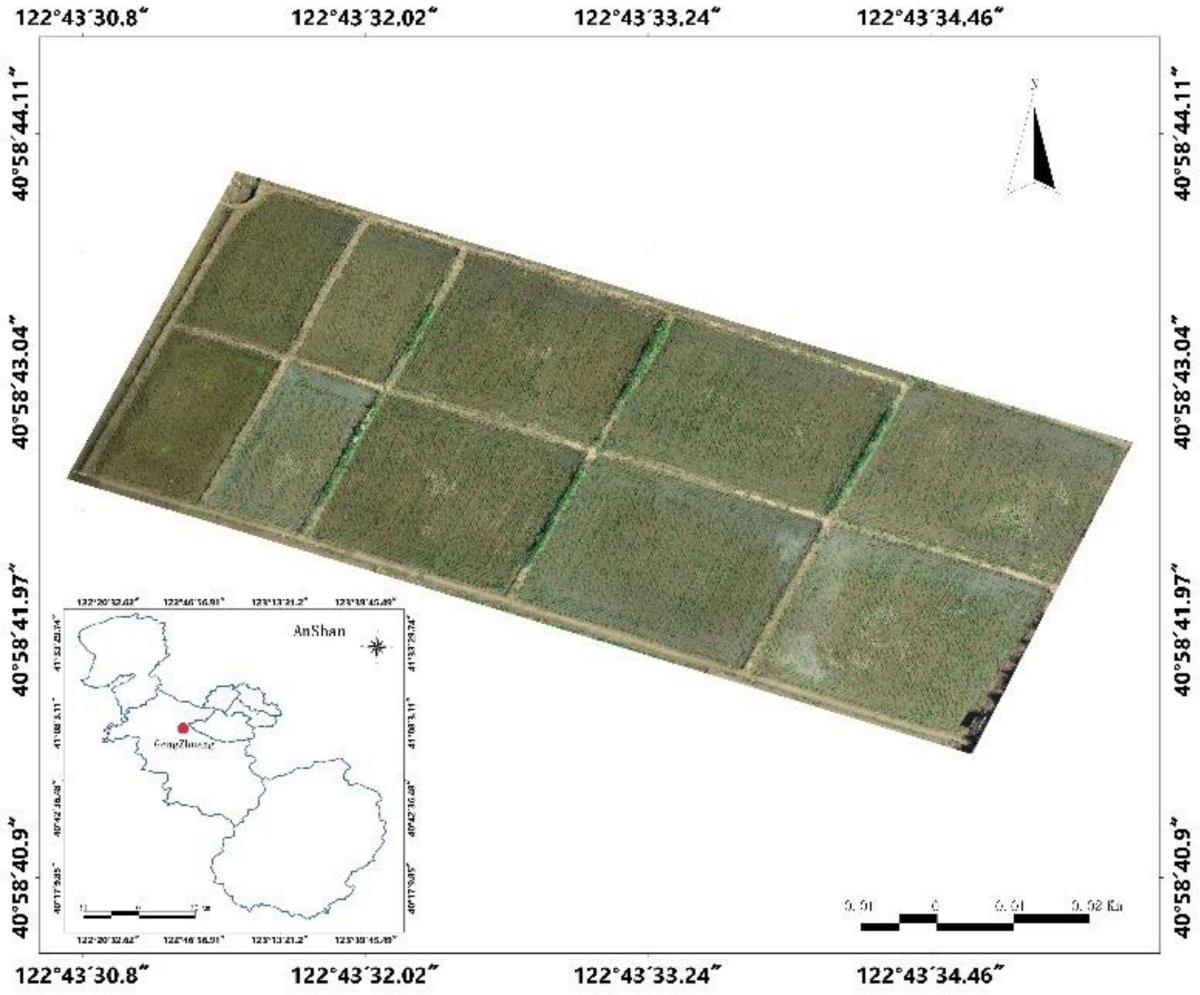
Study area.

The study area is located at the experimental base of Shenyang Agricultural University, Haicheng City, Liaoning Province (40°58′43″N, 122°43′33″E). It has a monsoon climate of medium latitudes with an average summer temperature of 20°C–28°C, average annual precipitation of 721.3 mm, and abundant groundwater resources. The climatic conditions at this site are suitable for rice growth. The study area is shown in Figure 1.

The experimental rice variety was selected from Mongolian rice, which is susceptible to rice blast, and the planting area was 0.39 hm^2^. The planting density was 17 cm between rows and 14 cm between plants. No pest control treatments were applied to the trial area, and other field management was normal. At the jointing stage, spores of the laboratory-grown rice blast pathogen were configured into a spore suspension at a 9 mg/100 ml concentration and inoculated for leaf blast at 17:00 on July 6, 2021. The inoculation method is manual spray inoculation. Firstly, the prepared spore suspension is shaken well and then evenly sprayed onto the surface of rice leaves until the leaf surface is completely covered with tiny droplets. After injection, a black and wet plastic bag was used for wrapping to maintain humidity and promote its onset, and the bag was removed at 7:00 am the next day. Also, the degree of rice leaf blast incidence is positively correlated with the number of inoculated rice in a specific range. Therefore, we inoculated 40 acres in the severe disease area, 20 in the moderate disease area, and 10 in the mild disease area. Rice in the inoculated area began to show symptoms of rice blast disease on July 11. Under the guidance of plant protection experts, healthy and diseased rice plants were randomly dug from the field every 5 days and brought back to the hyperspectral laboratory. And in the laboratory, leaf samples of different disease levels were collected to obtain hyperspectral image data. At the same time, the disease level classification refers to the GTB 15790-2009 rules of investigation and dorecast of the rice blast. The level classification criteria and data volume are shown in Table 1.

**Table 1:** Disease classification criteria and sample size statistics.

Disease levels	Disease classification criteria	Training sets	Testing sets
Level 0	No disease spots	48	22
Level 1	Disease spot area less than 1% of leaf area	49	19
Level 2	Disease spot area of 1%–5% of leaf area	55	20
Level 3	Disease spots covering more than 5% of the leaf area	49	23
Total	–	201	84

### Spectral Data Acquisition and Processing

#### Hyperspectral Image Acquisition and Correction

**Figure 2:**
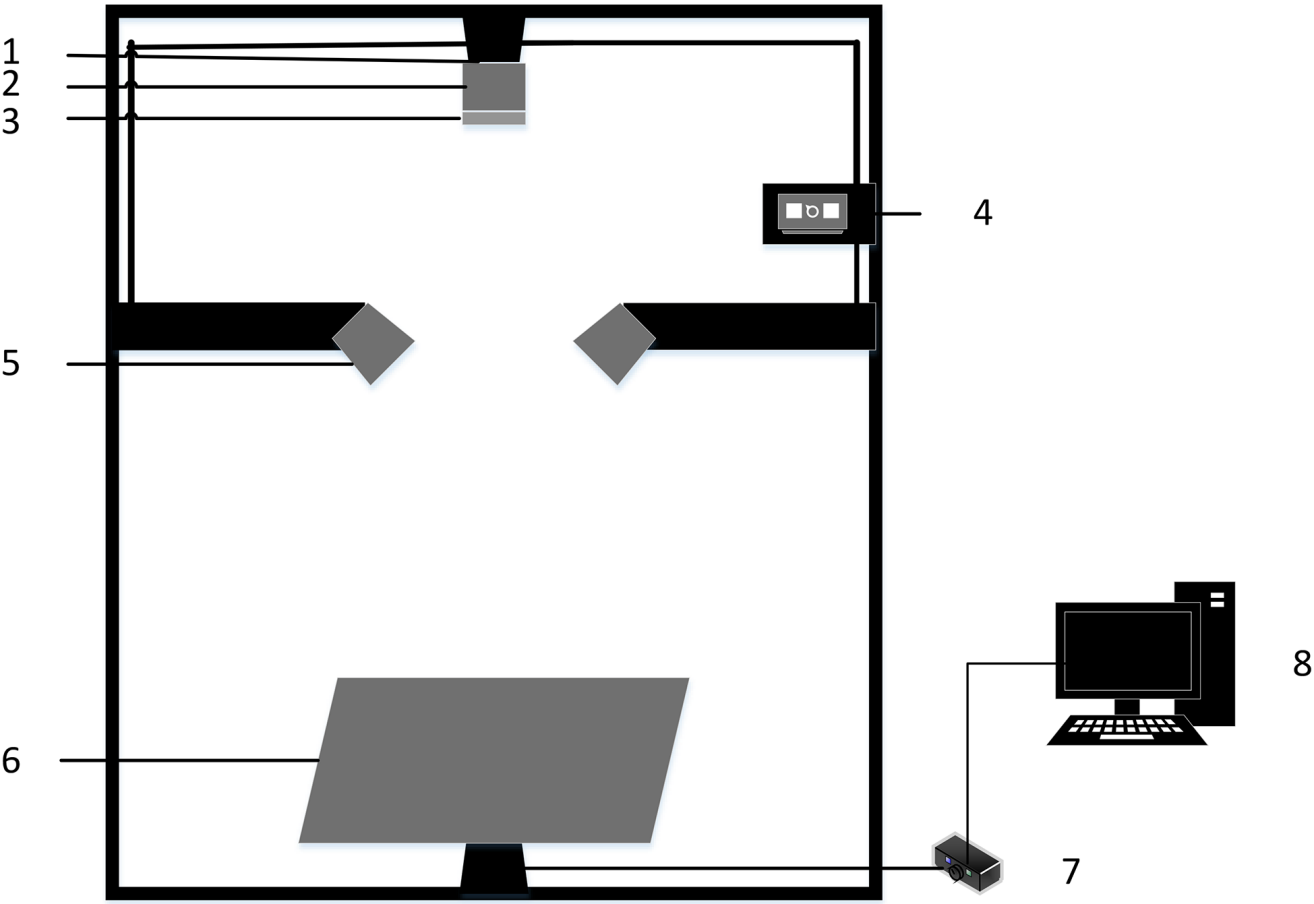
Hyperspectral imaging system: (1) CCD camera; (2) Hyperspectral imaging spectrometer; (3) Lens; (4) Light source controller; (5) Light source; (6) Displacement stage; (7) Displacement stage controller; and (8) Computer.

In this study, a hyperspectral imaging system was used to acquire hyperspectral images of rice leaves, as shown in Figure 2. The main components of the system include a hyperspectral imaging spectrometer (ImSpector V10E, Spectral Imaging Ltd., Finland), a CCD camera with a resolution of M1392*1040 (IGV-B1410M, IMPERX incorporated, United States), two 150 W fiber-optic halogen lamps (Ocean Optics, Dunedin, FL, United States), a precision displacement control stage (IRCP0076-1 COM, Taiwan), lightless dark box (120 cm × 50 cm × 140 cm), and computer (DELL Vostro 5560D-1528). The hyperspectral imaging system acquired spectra in the range of 400–1,000 nm with a spectral resolution of 0.64 nm.

To obtain hyperspectral image data of rice leaves with the best image quality, the hyperspectral imaging system needs to be adjusted. Adjust the left and suitable light sources so that the light emitted from them forms a straight line directly below the camera lens. And change the distance between the camera lens and the rice leaf sample to 30 cm. At the same time, the displacement stage movement speed was set to 1.2 mm/s to ensure image clarity. Before each acquisition of hyperspectral images, the acquisition instrument needs to be calibrated in black and white. Acquire the all-white calibration image RW of the hyperspectral image correction whiteboard and adjust the exposure time so that the value of Max DN reaches 80% of the maximum value (Max DN is set to 3,200). Use the lens cap to block the lens and acquire the all-black calibration image RD after securing the lens. When acquiring hyperspectral images of rice leaves, a group of 5–6 isolated rice leaves was laid flat to a pure black and opaque black background plate and placed on a displacement platform for spectral imaging. The exposure time was adjusted again so that the Max DN value still reached 3,200, and the images were captured using Spectra-Image acquisition software, and the data were saved. However, due to the influence of the state of the experimental equipment, the level of the test personnel, and other factors, the acquired hyperspectral images of the leaves often have some noise. Therefore, to mitigate the effect of noise on the study, the following equation was used for the correction process.


(1) $$R=\frac{I_{\mathrm{sample}}-I_{\mathrm{dark}}}{I_{\mathrm{white}}-I_{\mathrm{dark}}}$$

Where $I_{\mathrm{sample}}$ denotes the original hyperspectral reflectance of rice leaves, $I_{\mathrm{white}}$ denotes the image reflectance corrected by all-white, and $I_{\mathrm{dark}}$ denotes the image reflectance corrected by all-black, and R is the corrected hyperspectral image reflectance of rice leaves.

#### Hyperspectral Image Processing

**Figure 3:**
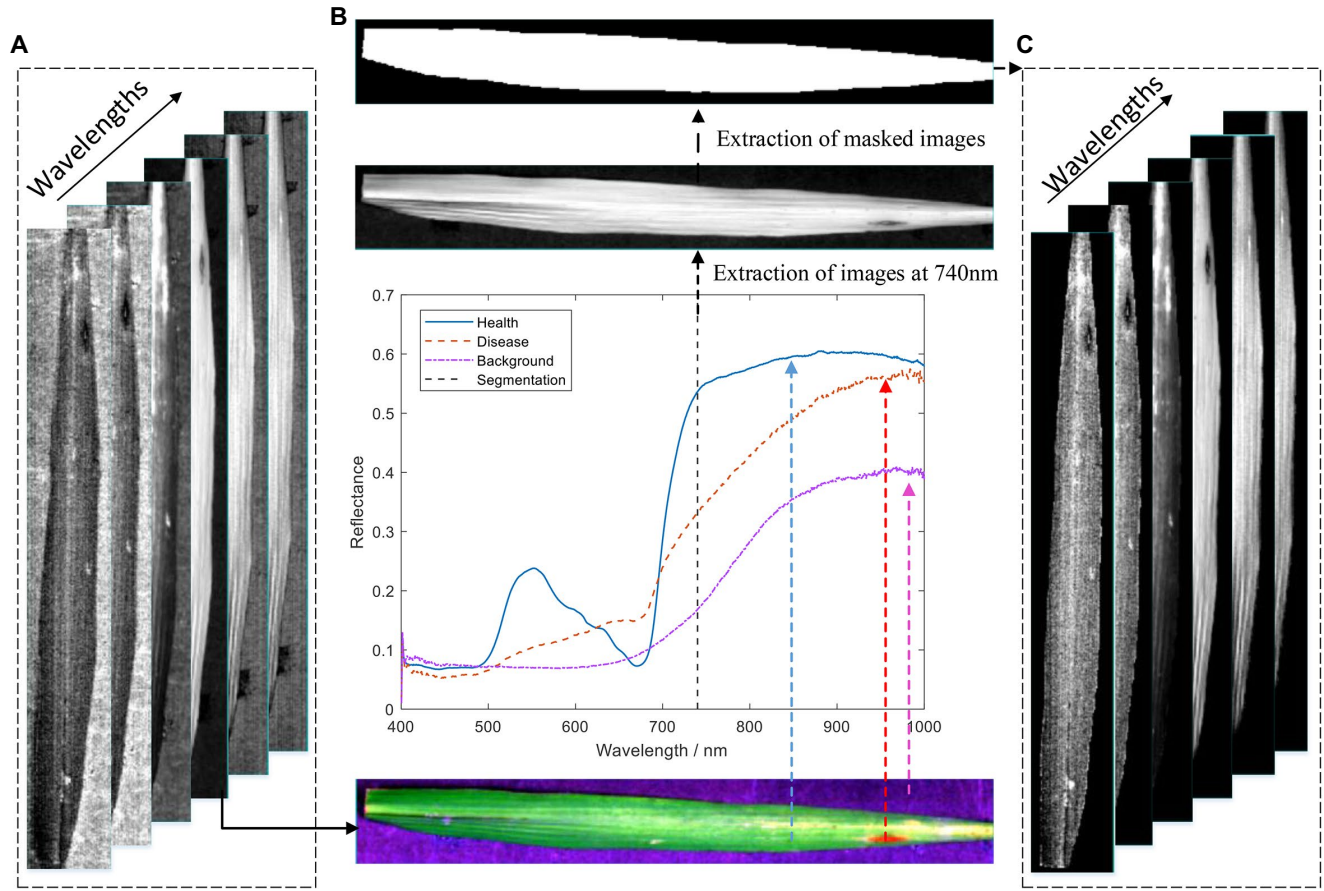
Flow chart of hyperspectral image data extraction: **(A)** original hyperspectral image; **(B)** acquisition of mask image; and **(C)** hyperspectral image of a leaf with background removed.

In the original hyperspectral image (Figure 3A), the spectral information of the leaves and the spectral information of the black background plate are included. Therefore, to reduce the background error, the acquired raw hyperspectral images need to be processed for removing the background and extracting the leaf regions. As shown in Figure 3B, there is a significant difference in the spectral reflectance between the black background plate and the healthy and diseased areas of the rice leaves. The comparative analysis shows that the difference in spectral reflectance between the background and leaf lesion areas and healthy areas at 740 nm is significant. Therefore, the 740 nm spectral image was extracted for extracting the mask. The best segmentation of the background and the leaf region was achieved through several tests when the segmentation threshold was set to 0.18. The masked image is shown in Figure 3B. Finally, the background of the original hyperspectral image is removed by the logic operation, and the result is shown in Figure 3C.

All 285 leaf sample images were subjected to the above processing to extract leaf regions in this study. Also, the whole leaves were treated as independent regions of interest, and the mean spectral reflectance was extracted using ENVI 5.3 (ITT Visual Information Solutions, Boulder, CO, United States). Meanwhile, the SG method was used for the denoising process to effectively improve the signal-to-noise ratio and reduce the effect of random noise. The processed spectral data were used as the original hyperspectral data of leaf samples with different rice blast disease classes.

### Disease Feature Extraction Method

#### Texture Feature Extraction

Texture features are inherent characteristics of the surface of an object, and they reflect the changes in the physical characteristics of the crop surface during crop growth. When a crop is infected with a disease, leaf pigmentation and internal structure changes can lead to leaf surface color and morphology, which can cause leaf texture features. Therefore, texture features can detect different levels of diseased leaves. This study obtained eight texture features based on gray level co-occurrence matrix (GLCM) to detect rice leaf blast. GLCM is a standard method to study the spatial correlation properties of grayscale to describe textures (Haralick et al., 1973; Tian et al., 2014). The method calculates the mean and SD of energy, contrast, entropy, and correlation from four directions, such as 0°, 45°, 90°, and 135°. However, due to a large amount of redundant information in the hyperspectral images, direct texture feature extraction will significantly impact the model accuracy. Therefore, the principal component analysis method is used to dimensionality reduce the hyperspectral images to obtain the principal component images containing a large amount of effective information (contribution rate greater than 95%). Then, the texture features are extracted from the obtained principal component images.

#### Characteristic Wavelength Screening

ElasticNet is a multiple linear regression algorithm with L1 and L2 norms as regularization matrices (Hui and Trevor, 2005). The algorithm achieves the screening of critical model variables by building a model to obtain L1 and L2 norms and using them as mixed penalty terms to compress the smaller coefficients obtained by the least squares method estimation to zero, thus getting a sparse solution. The ElasticNet method preserves the sparsity characteristics of the sample features by using L1 and L2 norms and gets the stability of the ridge regression L2 regularization during the loop iteration. The redundant variables are effectively eliminated in constructing the multivariate linear model, and the feature variables with high significance are retained.

Principal component analysis loading is a widely used algorithm for wavelength extraction of spectral features in spectroscopy (Zhao et al., 2016b). The advantage of this method is its ability to eliminate the problem of multicollinearity between spectral variables while retaining as much of the original spectral information as possible (Williams et al., 2010; Feng et al., 2020a). PCA loading was performed by the PCA method to screen the principal components with contribution rates greater than 95% and determine the loadings under the corresponding principal components. Then, the wavelengths corresponding to the wave peaks and troughs were screened by wavelength-loading graphs as the characteristic wavelengths of the disease.

Successive projections algorithm is a forward feature variable screening method (Araújo et al., 2001). This method uses the vector projection analysis method to project wavelengths in the spectrum onto other wavelengths, compare the magnitude of the two projection vectors, and obtain the wavelength with the largest vector as the sensitive wavelength to be selected. Then, the root-mean-square error (RMSE) of the model is obtained by building multiple linear regression calibration models validated by a separate test set. Among the wavelengths to be selected, the number of wavelengths corresponding to the minimum RMSE is chosen as well as the specific wavelength. This method can maximize the elimination of redundant information in the spectral data and minimize the covariance between variables (Zhang et al., 2019). It can also greatly reduce the number of variables used for modeling and improve the speed and efficiency of modeling.

### Modeling Methods

#### Adaptive-Weight Immune Particle Swarm Optimization ELM

Adaptive-weight immune particle swarm optimization ELM (AIPSO-ELM) is an improved algorithm proposed based on the ELM algorithm. Since the weights and biases of ELM are generated randomly, the weights and biases are not updated during the training process. It is found that this way of generating weights and biases in ELM is prone to inconsistent model results and a lack of explanations in the learning process. Therefore, this study proposes a hybrid algorithm combining adaptive-weight immune particle swarm optimization algorithm (AIPSO) and ELM model. The specific AIPSO optimization process is as follows.

Step 1: Initialize the particle-matrix, the individual extremum matrix, and the global extremum. Assume that *N* particles form a swarm in a D-dimensional weight and bias search space. A logistic regression analysis mapping generates an N*D-dimensional particle-matrix, where the i-th particle is represented as a D-dimensional vector.


(2) $$X_{i}=\left(x_{i1},x_{i2},x_{i3},\ldots ,x_{\mathrm{iD}}\right),i=1,2,\ldots ,N$$

At the same time, an N*D dimensional velocity matrix is randomly generated. The search velocity of the i-th particle is denoted as:


(3) $$V_{i}=\left(v_{i1},v_{i2},v_{i3},\ldots ,v_{\mathrm{iD}}\right),i=1,2,\ldots ,N$$

Step 2: Calculate the fitness value of the current particle population. The optimal position searched so far is called the individual optimal solution $P_{\mathrm{best}}$. Meanwhile, the optimal position searched by the whole particle swarm so far is the globally optimal solution $g_{\mathrm{best}}$.


(4) $$P_{\mathrm{best}}=\left(p_{i1},p_{i2},p_{i3},\ldots ,p_{\mathrm{iD}}\right),i=1,2,\ldots ,N$$

(5) $$g_{\mathrm{best}}=\left(p_{g1},p_{g2},p_{g3},\ldots ,p_{gD}\right)$$

Determine whether the maximum number of iterations is satisfied or the optimal solution has no change. If yes, end and output the result. Otherwise, proceed to step 3.

Step 3: Update the particle by updating its position and velocity with the following equation.


(6) $$v_{\mathrm{id}}=wv_{\mathrm{id}}+c_{1}r_{1}\left(P_{\mathrm{besti}}-x_{\mathrm{id}}\right)+c_{2}r_{2}\left(g_{\mathrm{bestd}}-x_{\mathrm{id}}\right)$$

(7) $$x_{\mathrm{id}}=x_{\mathrm{id}}+v_{\mathrm{id}}$$

Where w is the inertia weight, $c_{1}$ and $c_{2}$ are the learning factors, $r_{1}$ and $r_{2}$ are uniform random numbers in the range of [0,1]. Since the inertia weight w modulates the degree of change of the previous velocity on the current particle velocity, the inertia weight has a significant impact on the particle update. If *w* is overly large, the best position will be missed. If w is tiny, it will easily fall into the optimal local solution and not obtain the best particle position. Therefore, in this study, the inertia weight w is updated according to the distance of the global best point with the following equation.


(8) $$w=\{\begin{array}{c} w_{\text{min}}-\frac{\left(w_{\text{max}}-w_{\text{min}}\right)\times \left(f-f_{\text{min}}\right)}{f_{avg}-f_{\text{min}}},f\leq f_{avg}\\ w_{\text{max}},f>f_{avg}\; \end{array}$$

Where f denotes the objective function value of the particle, $f_{avg}$ and $f_{\text{min}}$ denote the average and minimum objective values of all particles, respectively. Therefore, the inertia weight w changes with the change of the accurate function value. When the particle target values are more dispersed, decrease w. When the particle target values are more concentrated, increase w.

Step 4: Generate immune memory particles, and generate *M* new particles by logistic mapping.

Step 5: Calculate the difference in fitness of each of the M + N particles in the current population.


(9) $$\mathrm{Density}\left(x_{i}\right)=\frac{1}{\sum_{j=1}^{N}|f\left(x_{i}\right)-f\left(x_{j}\right)|}\;i=1,2,3,\ldots ,N$$

Where $x_{i}$ denotes particle i and $f\left(x_{i}\right)$ is the value of the fitness function of particle i.

Step 6: Calculate the probability of producing N + M particles using the percentage of similar particles in the population. Select the *N* new particles with a higher likelihood to form a new population. Perform step 2 again.


(10) $$\begin{array}{l} P\left(x_{i}\right)=\frac{\frac{1}{Density\left(x_{i}\right)}}{{\sum^{\text{​}}}_{i=1}^{N}\frac{1}{Density\left(x_{i}\right)}}=\frac{{\sum^{\text{​}}}_{j=1}^{N}\left|f\left(x_{i}\right)-f\left(x_{j}\right)\right|}{{\sum^{\text{​}}}_{i=1}^{N}{\sum^{\text{​}}}_{j=1}^{N}\left|f\left(x_{i}\right)-f\left(x_{j}\right)\right|}\;i\\ \;=1,2,3,\ldots ,N \end{array}$$

It can be seen that the magnitude of the concentration selection probability of particle i depends on the number of particles that are similar to particle i. When the number of particles with high similarity to particle i in the population is high, the concentration of particle i increases, and the probability of particle i being selected decreases. Conversely, the probability of particle i being determined increases. This particle selection mechanism based on the similarity between particles can effectively ensure the diversity of particles in the algorithm and avoid premature convergence of the particle population, which significantly improves the solution accuracy and convergence speed in the later algorithm stage.

#### Other Modeling Methods

Support vector machine is a supervised classification learning algorithm that excels in data classification problems with small samples, nonlinearities, and high-dimensional feature spaces. The algorithm uses the risk minimization principle to reduce the upper bound on the model error while minimizing the error in the sample data (Xia et al., 2016). The SVM method splits the sample data by finding a hyperplane and converts the classification problem into solving a convex quadratic programming problem using an interval maximization learning strategy (Romer et al., 2011). The classification problems are usually nonlinear, and the hyperplane cannot be obtained directly in the low-dimensional space. Therefore, SVM cleverly transforms the nonlinear classification problem in the low-dimensional area into a linear classification problem in high-dimensional space by kernel function to solve the problem of linear indistinguishability in low-dimensional space.

Extreme learning machine (ELM) is a typical single implicit layer feed-forward neural network model consisting of the input layer, implied layer, and output layer (Huang et al., 2006). The algorithm randomly generates the continuous weight values between the input and hidden layers and the bias values of the neurons in the hidden layer. The optimal solution can be obtained without adjustment during the training process (Sun et al., 2014). Compared with traditional training methods, it has the advantages of fast learning speed. It is less prone to overfitting, which has attracted the research attention of a wide range of experts and scholars.

### Evaluation Metrics

To assess the accuracy of the developed model for early detection of leaf blast, overall accuracy (OA), kappa coefficient, precision, and recall were used as indicators for evaluating the model. Also, all of these indicators can be calculated from true positive (TP), true negative (TN), false positive (FP), and false negative (FN), as defined in Table 2. The specific formulae for calculating TP, TN, etc. are as follows.


(11) $$OA=\frac{TP+TN}{TP+TN+FP+FN}$$

(12) $$p_{e}=\overset{m}{\underset{i=1}{\sum }}\frac{\left(TP_{i}+FN_{i}\right)\left(TP_{i}+FP_{i}\right)}{N^{2}}$$

(13) $$\mathrm{Kappa}=\frac{OA-p_{e}}{1-p_{e}}$$

(14) $$\mathrm{Precision}=\frac{TP}{TP+FP}$$

(15) $$\mathrm{Recall}=\frac{TP}{TP+FN}$$

**Table 2:** Table of confusion matrix definitions.

	Positive	Negative
Positive	True Positive (TP)	False Negative (FN)
Negative	False Positive (FP)	True Negative (TN)

## Results and Analysis

### Spectral Curve Analysis of Different Disease Levels

**Figure 4:**
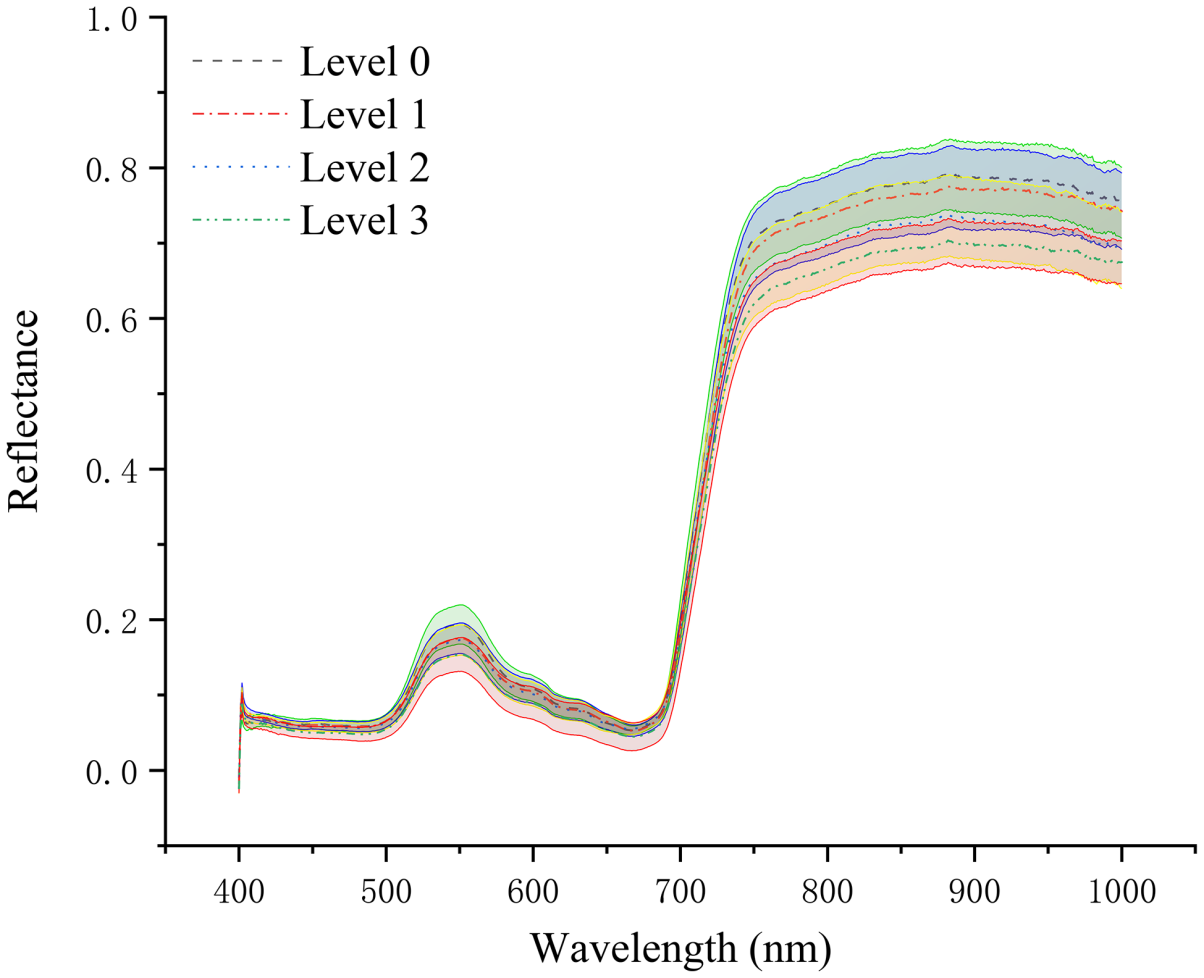
Average leaf reflectance curves and corresponding standard deviation values of healthy rice plants and rice plants at different disease stages.

As shown in Figure 4, the spectral reflectance curves of healthy rice leaves (Level 0) and rice leaves infected with different degrees of leaf blast had similar trends. The mean spectral reflectance of healthy leaves was relatively high, the mean spectral reflectance of diseased leaves was the lowest, and the mean spectral reflectance of level 1 and level 2 infected leaves was somewhere in between. Among them, in the visible region (450–680 nm), the average spectral reflectance curves of levels 1 and 2 nearly overlap and are smaller than the average spectral reflectance of healthy leaves. However, in the NIR region, significant spectral differences in the spectral reflectance of different disease levels emerged. The spectral reflectance of healthy leaves was slightly greater than that of level 1 diseases and significantly greater than the spectral reflectance of level 2 and 3 infections. The reason for this is that the pathogens infest the chloroplasts and cell walls of the leaf cells causing damage to the cell structure, reducing the multiple reflections of light between the cells, which contributes to the decrease in spectral reflectance. At the same time, the coefficient of variation (CV) for each disease class is less than 0.15 (Table 3), indicating a degree of variability in spectral reflectance. It can be seen from the figure that in the wavelength range of 400–450 nm, there is noise and obvious overlap between the spectral data of the diseased and normal regions of rice. Therefore, in order not to affect the modeling accuracy, the spectral data in the range of 450–1,000 nm were selected for this study’s follow-up study.

**Table 3:** The Coefficient of variation of spectra of different disease levels.

Level	Level 0	Level 1	Level 2	Level 3
CV	0.0702	0.0771	0.0810	0.0642

### Analysis of Dimensionality Reduction Results

#### PCA Loading Screening Characteristic Wavelength

**Figure 5:**
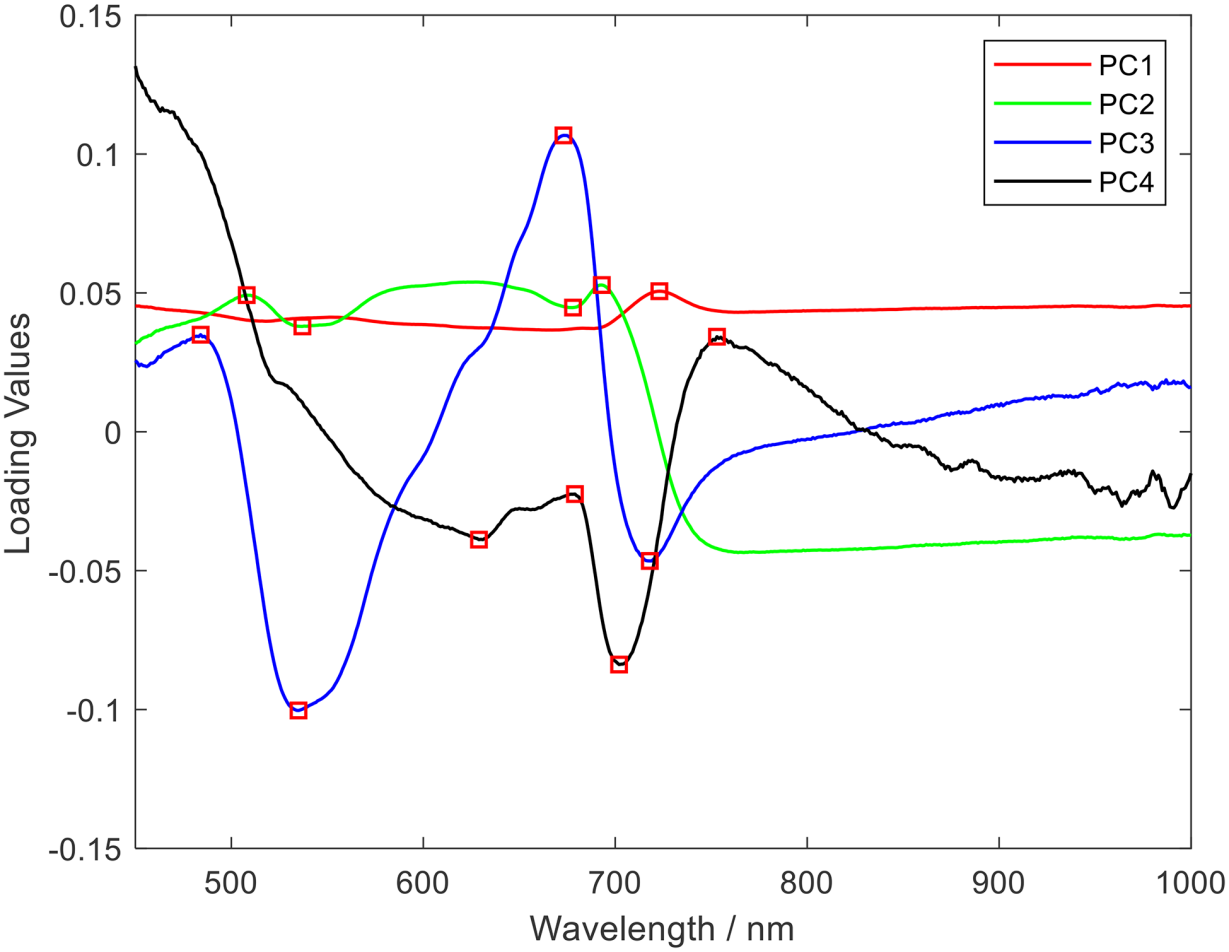
Selected o spectral characteristics wavelength using principal component analysis (PCA) loading (The wavelength of the spectrum in red is the selected characteristic wavelength).

The PCA algorithm was used to reduce data dimensionality on the hyperspectral data to obtain the principal components with the most significant correlation with the spectral wavelength variables. As shown in Table 4, the cumulative contribution of the first four principal components was 99.56%, which was greater than 99%, by PCA dimensionality reduction. Also, the loading coefficients of the first four principal components were obtained. By plotting the loading coefficients of the first four principal components, the wavelengths where the peaks and troughs were located were accepted as the characteristic wavelengths of rice blast, as shown in Figure 5. The selection results are shown in Table 5.

**Table 4:** The cumulative contribution of some principal components.

PCs	Eigenvalure	Contribution/%	Accumulative contribution/%
1	373.74	67.83	67.83
2	157.20	28.53	96.36
3	14.52	2.63	98.99
4	3.12	0.57	99.56
5	1.12	0.20	99.76

**Table 5:** Spectral characteristic wavelengths for ElasticNet, PCA, and SPA screening.

Descending dimension method	Characteristic wavelengths/nm
ElasticNet	534,681,682,684,686,687,749,752,754,768,982,984,993, and 995
PCA	484,508,835,837,629,673,678,679,693,702,718,723, and 753
SPA	497,533,580,659,681,704,735,841,990, and 1,000

#### SPA Screening Characteristic Wavelength

**Figure 6:**
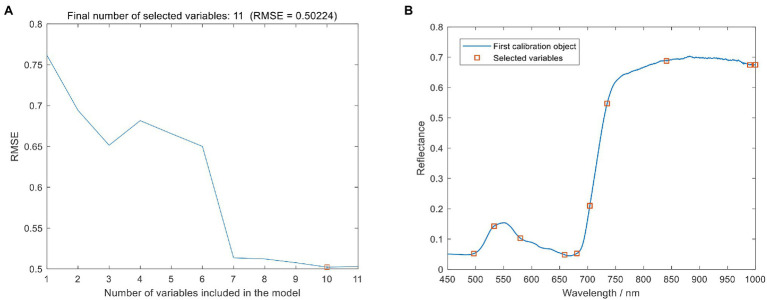
Selected o spectral characteristics wavelength using sequential projection algorithm (SPA). **(A)** Number of optimal spectral variables. **(B)** Characteristic wavelengths for screening.

The SPA was used to dimensionality reduce the spectral data to obtain the spectral characteristic wavelengths that can effectively express the information of leaf blast. In this study, the minimum and maximum screening wavelength numbers were set to 5 and 12, respectively, and the RMSE was used as the evaluation criterion, as shown in Figure 6. During the screening process of SPA, the RMSE value showed a gradual decrease with the gradual increase of the number of selected wavelengths. When the wavelengths were 10, the RMSE decreased to the lowest value (RMSE = 0.50224). Therefore, the final SPA screened 10 optimal spectral wavelengths, as shown in Figure 6B with Table 5.

#### ElasticNet Screening Characteristic Wavelength

**Figure 7:**
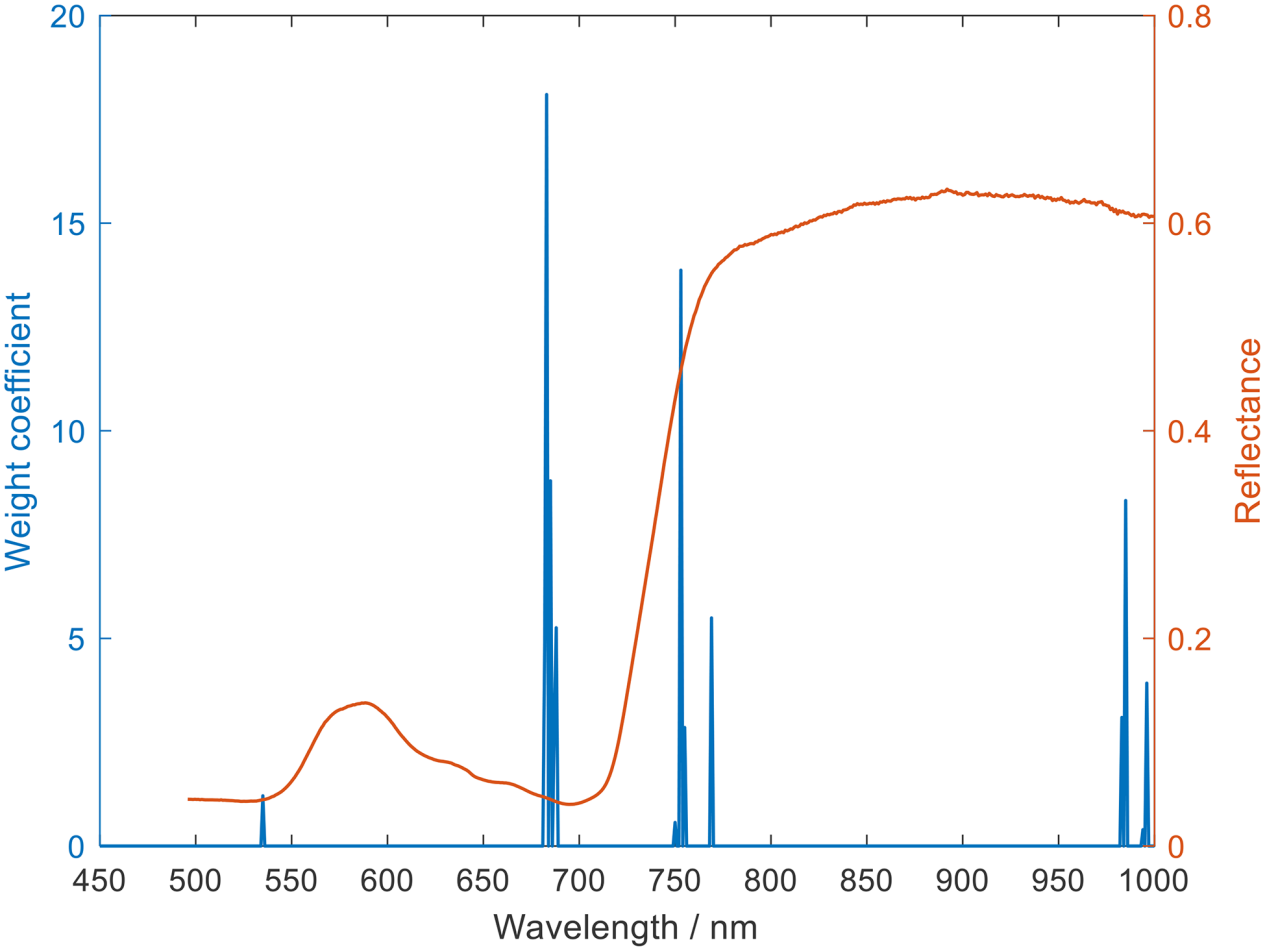
Selected o spectral characteristics wavelength using ElasticNet.

The ElasticNet method can effectively perform the selection and dimensionality reduction of characteristic wavelengths in the spectral analysis while reducing the correlation between spectral wavelengths. In this study, the l1_ratio and alphas were set to 1 and 0.005, respectively. And a 5-fold cross-validation was performed, and the dimensionality reduction results are shown in Figure 7. The ElasticNet method reduced the spectral wavelengths from 551 to 14 dimensions, accounting for about 2.54% of the full wavelengths, as shown in Table 5. The filtered characteristic wavelengths are uniformly distributed in the visible, red-edge, and near-infrared regions.

### Texture Feature Screening

The GLCM method extracts texture features from hyperspectral principal component images. And the accuracy of the GLCM algorithm depends on two critical parameters: the relative pixel distance D measured in the number of pixels and the relative orientation θ. In this study, we set the close pixel distance *D* = 1 and calculate the texture features such as energy and entropy from four directions, such as 0, 45, 90, and 135°, respectively. Finally, we calculate their mean and SD, respectively. This study conducted the Pearson correlation analysis to screen the best texture features for eight texture features, including energy average, energy SD, and entropy average, to eliminate redundant texture features further and improve modeling accuracy. The results are shown in Table 6.

**Table 6:** Results of the Pearson correlation analysis.

Texture features	*p*	Significance
Energy-Avg	0.829	**
Energy-Std	−0.397	**
Entropy-Avg	−0.576	**
Entropy-Std	−0.548	**
Contrast-Avg	−0.345	**
Contrast-Std	−0.376	**
Correlation-Avg	−0.040	–
Correlation-Std	−0.201	**

“**” indicates a significant correlation at 0.01. “-” indicates no significant correlation. Energy-Avg, Energy-Std represents the capacity mean and energy SD, respectively, the same below.

As shown in Table 6, the average and SD of energy, entropy, and contrast showed highly significant correlation with different disease levels of leaf blast among the eight textural features. And the average value of correlation was poorly correlated with the various disease levels of leaf blast. The value of *p* of correlation average with disease level was −0.040, and showed no correlation. Therefore, the texture features most sensitive to rice leaf blast (average and SD of energy, average and convenient deviation of entropy, average and SD of contrast, and SD of correlation) were used in this study to build a classification model for accurate identification of rice leaf blast.

### AIPSO-ELM Modeling Analysis

Single spectral features (ElasticNet, PCA, and SPA), texture features, and fusion features were used to construct the AIPSO-ELM leaf blast classification model, respectively, and the results are shown in Table 7. The classification accuracy of the models built with the spectral wavelengths selected by SPA, ElasticNet, and PCA loading methods was better in modeling using single spectral features. Compared with PCA and SPA, the models constructed based on characteristic wavelengths screened by ElasticNet were able to identify different levels of leaf blast more accurately, with OA and Kappa of 94.05 and 92.05%, respectively. When only texture features were used for modeling, good classification results were also achieved with OA and Kappa greater than 88%, indicating that disease image texture features can effectively classify the disease. Compared with the modeling results of single features, the classification accuracy of the fusion feature-based classification model was significantly improved, with both OA and Kappa greater than 94 and 92%. The fusion method of ElasticNet+TFs has the highest classification accuracy with 97.62 and 96.82% for OA and Kappa, respectively. Compared with the results of ElasticNet and TFs, OA and Kappa improved by 3.57, 9.52, 4.77, and 12.72%, respectively. The classification results for different disease levels showed that the precision and recall of ElasticNet+TFs were 100% for levels 0 and 3 and 90.48 and 90.00% for levels 1 and 2 disease classification, respectively. This result indicates that the model constructed using fused features has high recognition accuracy for rice leaf blast.

**Table 7:** Modeling results of AIPSO-ELM algorithm.

Features	Precision/%				Recall/%				OA/%	Kappa/%
	Level 0	Level 1	Level 2	Level 3	Level 0	Level 1	Level 2	Level 3		
ElasticNet	95.65	94.12	86.36	100.00	100.00	84.21	95.00	95.65	94.05	92.05
PCA	100.00	100.00	76.92	100.00	100.00	78.95	100.00	91.30	92.86	90.46
SPA	95.65	93.75	81.82	95.65	100.00	78.95	90.00	95.65	91.67	88.86
TFs	86.96	82.35	82.61	100.00	90.91	73.68	95.00	91.30	88.10	84.10
ElasticNet+TFs	100.00	90.48	100.00	100.00	100.00	100.00	90.00	100.00	97.62	96.82
PCA + TFs	100.00	90.48	94.74	95.65	95.45	100.00	90.00	95.65	95.24	93.64
SPA+TFs	94.45	85.00	94.74	100.00	95.45	89.47	90.00	100.00	94.05	92.05

### Modeling Results of Other Classification Models

To further determine the best disease characteristics and the best classification model, we compared the AIPSO-ELM model with the SVM and ELM models for analysis, respectively. The classification results of SVM and ELM are shown in Table 8.

**Table 8:** Modeling results of SVM and ELM.

Model	Features	Precision/%				Recall/%				OA/%	Kappa/%
		Level 0	Level 1	Level 2	Level 3	Level 0	Level 1	Level 2	Level 3		
SVM	ElasticNet	100.00	76.19	85.00	95.45	95.45	84.21	85.00	93.33	89.26	85.71
	PCA	100.00	75.00	80.95	96.65	90.91	78.95	85.00	95.65	88.10	84.11
	SPA	100.00	73.68	77.27	95.65	90.91	73.68	85.00	95.65	86.90	82.52
	TFs	85.71	76.19	90.00	95.65	81.82	84.21	90.00	91.67	87.06	82.72
	ElasticNet+TFs	95.45	94.44	95.24	100.00	95.45	89.47	100.00	100.00	96.43	95.23
	PCA + TFs	91.67	100.00	90.91	100.00	100.00	89.47	100.00	91.30	95.24	93.64
	SPA+TFs	95.24	90.00	95.24	100.00	93.02	92.31	97.56	97.78	95.24	93.65
ELM	ElasticNet	95.00	77.27	81.82	100.00	86.36	89.47	90.00	86.96	88.20	84.14
	PCA	100.00	83.33	69.23	100.00	95.45	78.95	90.00	82.61	86.75	82.55
	SPA	100.00	77.27	76.19	90.48	90.91	89.47	80.00	82.61	85.75	80.96
	TFs	94.44	75.00	75.00	86.36	77.27	94.74	75.00	82.61	82.40	76.22
	ElasticNet+TFs	90.91	89.47	86.96	95.00	90.91	89.47	100.00	82.61	90.75	87.30
	PCA + TFs	87.50	93.33	80.00	100.00	95.45	73.68	100.00	86.96	89.02	85.69
	SPA+TFs	90.91	88.24	83.33	90.48	90.91	78.95	100.00	82.61	88.12	84.11

As shown in Table 8, both SVM and ELM models achieved better classification results. Similarly, the classification models constructed by fusing features obtained higher classification accuracy than single features. The classification model built with ElasticNet+TFs as input has the highest accuracy, with OA and Kappa greater than 90 and 87%, respectively. And compared with the AIPSO-ELM model proposed in this paper, the modeling results of both SVM and ELM are worse. Compared with the SVM model, the OA and Kappa of ElasticNet+TFs combined with the AIPSO-ELM model are improved by 1.19 and 1.59%, respectively. And compared to the ELM model, the OA and Kappa of ElasticNet+TFs combined with the AIPSO-ELM model enhanced by 6.87 and 9.52%, respectively. Meanwhile, in the comparative analysis with ELM, it can be further shown that the optimization method proposed in this study can substantially improve the classification performance of the ELM model. In addition, in the comparative analysis of the models built by single features and fused features; it can be shown that the fused features are more effective in identifying rice leaf blast and improving the identification accuracy.

## Discussion

Leaf blast disease poses a significant threat to rice production and can directly cause rice yield reduction or even crop failure. Efficient and accurate detection of leaf blast occurrence and timely control is essential to improve rice yield and quality (Yang et al., 2014). Hyperspectral imaging technology is currently an actual means of non-destructive disease detection. It has gradually attracted widespread attention from scholars at home and abroad because of its rapid, nondestructive, and accuracy (Sankaran et al., 2010; He et al., 2015). Therefore, we used a hyperspectral imaging system to obtain hyperspectral data of different disease levels and constructed rice leaf blast identification models from spectral characteristic wavelengths, texture features, and fusion features.

As can be seen from Figure 5, although the spectral curves of rice leaves of different disease levels showed approximately the same trend, the spectral differences between the spectra of healthy leaves and diseased leaves could still be seen. First, in the 530–580 nm green light band, the internal pigmentation was destroyed due to the infection of rice leaves by the rice blast fungus, thus causing the spectral reflectance of the diseased leaves to be lower than the spectral reflectance of healthy leaves. This phenomenon is consistent with the findings of Yuan et al. (2016) in their study on leaf blast discrimination. And the spectral reflectance of different disease classes overlapped more severely in the red band and red-edge band range. At the same time, no significant reflectance changes caused by diseases were observed in these two band ranges. Second, there was a significant spectral variation between healthy and infected diseased leaves in the 780–1,000 nm range. Healthy leaves had higher spectral reflectance values in the near-infrared than diseased leaves due to the invasion of the pathogen that severely damaged the cell structure and intracellular nutrient content. The same conclusion was given in the previous literature. The presence of these differences offers the possibility of identifying rice leaves with different grades of leaf blast. At the same time, during the infestation process, the pathogen will change the leaf’s internal structure and the external morphological characteristics of the leaf. The spectral reflectance is more sensitive to the change of the interior features of the plant, which is considered a “probe” for the detection of plant diseases (Al-Saddik et al., 2018). The evolution of external morphological characteristics can also be an essential basis for plant disease detection (Dang et al., 2020). Therefore, in this study, we extracted spectral characteristic wavelengths and texture features from the spectral and pixel dimensions of the imaging data, respectively, which are highly relevant to leaf blast disease to construct a classification model.

In terms of disease spectral feature extraction, spectral data dimensionality reduction is mainly divided into two types of feature extraction and wavelength screening. However, since the feature extraction methods are obtained by a combination of linear or nonlinear methods, the physical information of the original spectrum is corrupted to some extent (Zhao et al., 2020a). The wavelength screening method can directly screen the characteristic wavelengths associated with the disease from the spectral dimension, which can reduce the data dimension and reduce the correlation between wavelengths (Zhao et al., 2020b). In many studies, many researchers have used PCA-loading, SPA, and ElasticNet methods for data dimensionality reduction (Zhao et al., 2014; Yao et al., 2019; Zhang et al., 2020). Therefore, three methods, PCA loading, SPA, and ElasticNet, were used to screen the acquired full-band data for spectral characteristic wavelengths, respectively. As shown in Table 5, PCA loading, SPA, and ElasticNet selected characteristic wavelengths of 2.36, 1.82, and 2.55% of all wavelengths, respectively, which significantly reduced the data dimensionality. As for the extraction of image texture features, there are mainly statistical methods, geometric methods, and model methods. And in the previous studies on diseases, scholars mostly used the GLCM of statistical methods for texture feature extraction (Manivannan et al., 2012; Al-Sahaf et al., 2017). Therefore, in this study, eight texture features such as energy mean and energy SD in the images were obtained by GLCM. Meanwhile, the Pearson correlation analysis was used to select the texture features further, and the analysis results are shown in Table 6. The last seven features with high significance with the disease level were obtained as the final texture features. Therefore, in this study, disease features obtained from PCA loading, SPA, ElasticNet, and GLCM and their fusion features were used as model inputs to construct disease early detection models to determine the best disease detection features.

Some studies have shown that machine learning algorithms can accurately detect crops, such as rice (Kang et al., 2021), wheat (Yang et al., 2013), soybean (Nagasubramanian et al., 2019), and corn (Del Fiore et al., 2010). SVM is one of the more commonly used methods in machine learning, especially in qualitative research. SVM is one of the more widely used methods in machine learning, especially in qualitative research. For example, disease detection (Jin et al., 2013), weed detection (Papp et al., 2021), and terrain classification (Li et al., 2020b). And because hyperspectral images contain a large amount of information and have a sizeable sparse data structure, researchers often use SVM methods as the primary modeling tool. This study constructed seven early leaf blast SVM detection models using the disease features obtained from PCA loading, SPA, ElasticNet, and GLCM and their fusion features as model inputs. Overall the results were not as expected, with OA greater than 86% and Kappa greater than 82%. It may be due to the little variability of the early disease features, and the SVM did not find a good segmentation surface when converting the data space to a high-dimensional space.

Extreme learning machine is another machine learning algorithm used in this study. In previous studies, the ELM algorithm has been used more often to study diseases, such as tobacco (Zhu et al., 2017), tomato (Xie et al., 2015), and tea (Lu et al., 2020), but it has not been seen much in the early detection of rice leaf blast. In this study, the modeling analysis revealed that the ELM constructed by combining spectral features and texture features achieved the highest early detection accuracy (90.75% for OA and 87.30% for Kappa). However, the best detection accuracy was not achieved, and there were still more misclassification results. According to previous studies, the reason for the above phenomenon is that the weights and biases of the ELM model cannot be trained and updated (Feng et al., 2019; Yu et al., 2020). Thus the model cannot obtain the optimal solution. Therefore, getting the best weights and biases is the key to solving this problem.

**Figure 8:**
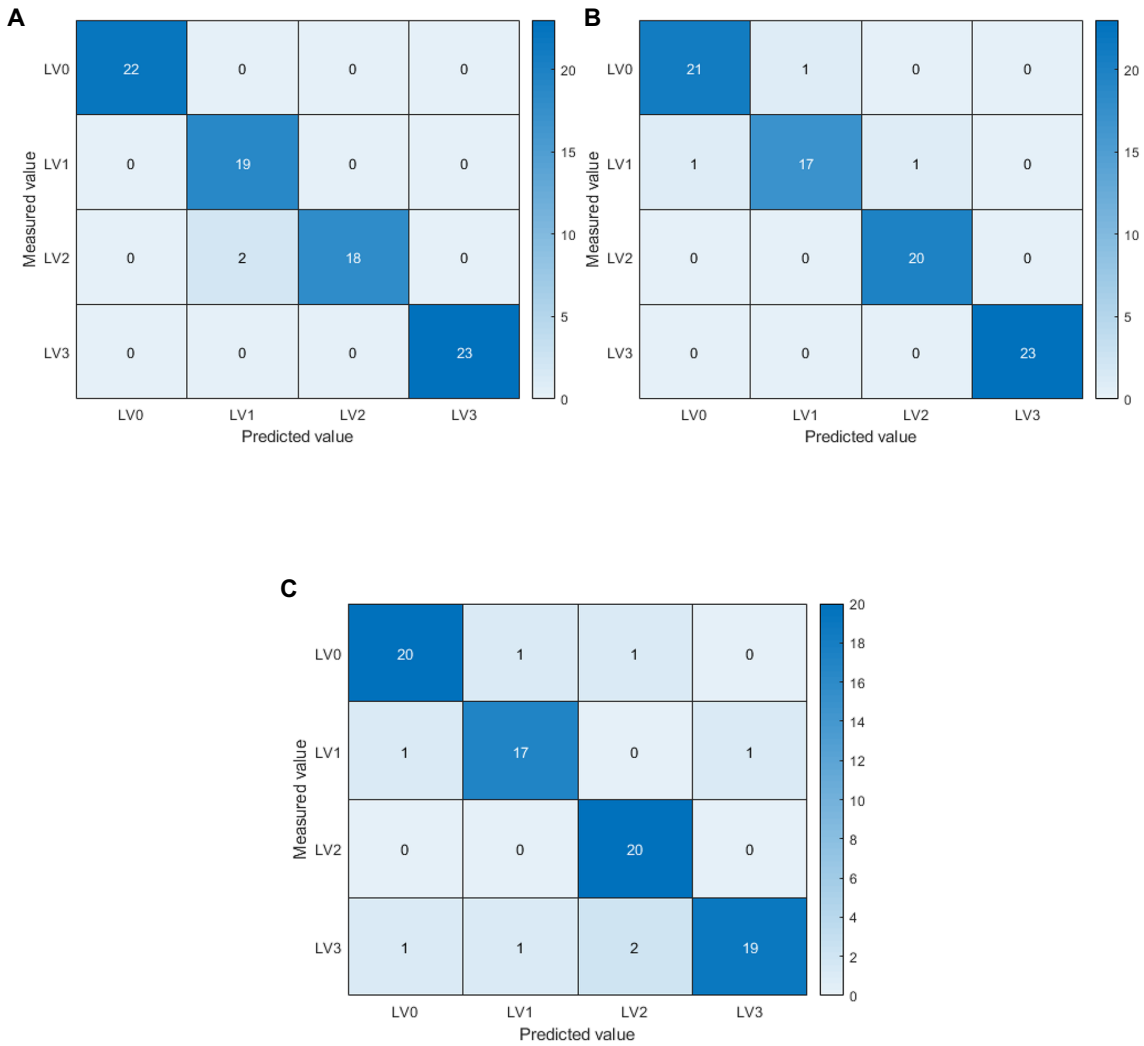
Confusion matrix based on ElasticNet+TFs modelling. LV0: Healthy (no disease spot); LV1: Level 1 (disease spot area less than 1% of leaf area); LV2: Level 2 (disease spot area of 1%–5% of leaf area); LV3: Level 3 (disease spots covering more than 5% of the leaf area). The diagonal line is the number of samples judged to be correct. **(A)** ElasticNet+TFs-adaptive-weight immune particle swarm optimization extreme learning machine (AIPSO-ELM), **(B)** ElasticNet+TFs-support vector machine (SVM), and **(C)** ElasticNet+TFs-ELM.

For the early detection of diseases, the problem of insignificant differences between disease features, and the problem of obtaining the best ELM weights and biases, this paper proposes an ELM classification model based on adaptive-weight immune particle swarm optimization. Meanwhile, this paper proposes an ELM classification model based on the AIPSO optimization algorithm to improve classification accuracy further. The AIPSO-ELM classification model was constructed using single disease features and their fusion features as model inputs. It was also compared with SVM and ELM models for analysis. The results are shown in Tables 7, 8. In terms of single disease feature modeling, the three classification models constructed with spectral characteristic wavelength and texture features achieved more desirable classification accuracy, with average OA and Kappa greater than 82 and 76%, respectively. The model’s classification constructed with the characteristic wavelengths screened by ElasticNet was better than the disease features obtained by SPA, PCA loading, and GLCM. ElasticNet-AIPSO-ELM had the highest classification accuracy with 94.05 and 92.05% for OA and Kappa, respectively. And on modeling with fused disease features, the classification model built by fused features has higher classification accuracy compared to single disease features (Knauer et al., 2017; Feng et al., 2022). This indicates that fusing spectral characteristic wavelengths with texture features can better represent the valid information contained in the disease images. Among them, ElasticNet+TFS-AIPSO-ELM model has the highest classification accuracy; OA and Kappa are 97.62 and 96.82%. As shown in Figure 8, in the specific disease class classification, the AIPSO-ELM model was misclassified only in the level 1 and level 2 classifications. The precision and recall rates were 100% for levels 0 and 3 and relatively low in the level 1 and level 2 disease classifications, with precision and recall rates of 90.48 and 90.00%. The other two models have more serious misclassification. In the fused feature modeling (ElasticNet+TFs), the AIPSO-ELM model has considerably improved classification effect over ELM and SVM models, with OA and Kappa improving by 6.87, 1.19, and 9.52%, 1.59%. This may be because the diversity of the immune particle populations during the training process drives the model to obtain better weights and biases. Also, the adjustment of adaptive inertia weights avoids falling into local optimal solutions, which significantly improves the model classification results.

In view of this, this study concluded from modeling and analysis of single disease features (spectral characteristic wavelengths and texture features) and fusion features that fusion features can better identify rice leaf blast disease. Meanwhile, in comparison with other models, it can be seen that the AIPSO-ELM model proposed in this study can provide accurate early detection of leaf blast and can provide a theoretical basis for the development of a portable multispectral imager for leaf blast.

In this part of the work, we only tested a single rice variety that was symptomatic for early disease detection and did not identify multiple rice varieties infected with rice blast but did not show symptoms. Therefore, in future work, we will conduct research on the early detection of undeveloped diseases in several rice varieties, leading to the effective prevention of rice leaf blast. At the same time, achieving early detection of rice leaf blast in the field is still more challenging. The difficulty in detecting the disease lies in external environmental factors, such as weather, light conditions, and background. While some environmental influences can be tested and analyzed under controlled conditions, the interaction of some influencing factors makes obtaining accurate spectral signatures of disease somewhat problematic. Therefore, a derivation method that enables high-quality indoor studies to be applied in a field setting is also a significant part of our following research.

## Conclusion

This study collected hyperspectral image data of rice leaves with different disease levels. The spectral characteristic wavelengths and texture features were screened using ElasticNet, PCA, SPA, and GLCM methods. The spectral characteristic wavelengths, texture features, and fused features were used to construct SVM, ELM, and AIPSO-ELM models. The results showed that the model’s classification accuracy based on fused features was significantly higher than that of single disease features. The model constructed by combining feature wavelengths screened by the ElasticNet method with texture features (ElasticNet+TFs) had the highest accuracy, with overall accuracy and Kappa coefficient more significant than 90 and 87%, respectively. Meanwhile, in terms of different modeling approaches, the AIPSO-ELM modeling approach proposed in this study obtained a more significant classification accuracy improvement compared to the SVM and ELM modeling approaches. In the fusion feature-based modeling (ElasticNet+TFs), AIPSO-ELM achieves the highest classification accuracy with 97.62 and 96.82% for OA and Kappa, respectively. Compared with ELM and SVM, the improvement is 6.87, 1.19, and 9.52%, 1.59% on OA and Kappa, respectively. This result proves that the AIPSO-ELM model method has a good application prospect in disease classification and helps improve the accuracy of disease level detection.

## Data Availability Statement

The raw data supporting the conclusions of this article will be made available by the authors, without undue reservation.

## Author Contributions

DZ and SF conceived and designed the experiments. DZ, SF, QG, and JL performed the experiments. DZ and GZ processed the raw data. DZ wrote the manuscript. YC, FY, and TX reviewed and edited the original manuscript. All authors contributed to the article and approved the submitted version.

## Funding

This work was supported by the Liaoning Provincial Key R&D Program Project (No. 2019JH2/10200002).

## Conflict of Interest

The authors declare that the research was conducted in the absence of any commercial or financial relationships that could be construed as a potential conflict of interest.

## Publisher’s Note

All claims expressed in this article are solely those of the authors and do not necessarily represent those of their affiliated organizations, or those of the publisher, the editors and the reviewers. Any product that may be evaluated in this article, or claim that may be made by its manufacturer, is not guaranteed or endorsed by the publisher.
